# Report of a Case of Hydrophobia, Which Occurred in Louisville, Ky.

**Published:** 1849-09

**Authors:** R. C. Hewett


Art. II.—*Report of a Case of Hydrophobia, which occurred in Louisville, Ky.* By R. C. Hewett, M. D.

The perfect mystery that has always surrounded cases of hydrophobia and the absolute absence of anything like known remedial means, coupled with the fact that the following is the only instance that has occurred in Louisville during the last six or seven years, justify a record and preservation of all the facts and phenomena that could be observed in the case. Though there may be no fact developed that can lead to valuable results, it must still be interesting to medical men to look at the terrible features of this awful malady, in every new case that may appear, to see if perchance some clew may be discovered by which its obscurity may be unshrouded.

The subject of this report was a patient of Dr. G. W. Thum, who attended him assiduously to the close, with Drs. Donne, M. Thum, Flint, and Hewett in consultation. Numerous other physicians of the city saw the case during its progress. The autopsy was most skilfully conducted by Dr. T. G. Richardson, assisted by Mr. J. Bartlett.

July 25th, 1849. Robert Adams, laborer, aged about thirty-two, dark complexion, stout, healthy looking, in attempting to drive off a large dog that seemed to be fighting a number of others, was bitten by him on the third phalanx of the right middle finger. This was about ten weeks ago as near as can be ascertained. The dog was considered rabid by all who saw him, and was killed. (It will not be irrelevant to mention in this connection, that one of the dogs bitten by this one, gave unequivocal signs of madness on the 30th inst.) Three days after receiving the bite, the patient called on Dr. Thum, who applied lunar caustic freely to the wound, which penetrated to the bone. No further complaint was made until this morning, when he sent for Dr. T., who found him with slight pain in the right shoulder blade, throbbing, uneasy sensation in the occipital region, hot, dry skin, langour and indisposition for exertion. He had taken breakfast as usual. Nothing was said at this time about the hand or arm. An emetic and warm pediluvium were ordered. In the afternoon the Doctor was sent for in haste. He was informed that the patient drank water for the last time at noon but ate nothing; attempts to swallow the emetic produced difficult, spasmodic breathing, and similar effects attended an attempt to put his feet in the warm water. Beginning to suspect hydrophobia, inquiry was made about the bitten finger, when he learned that for two or three days previous there had been pain in the dorsum of the metacarpal bone corresponding to it. This pain extended gradually up the arm to the shoulder to such a degree that the limb had been suspended in a sling. He was offered water to drink but said he could not swallow it, and turned away in evident distress. Water poured from a pitcher near him affected him in a similar manner. The inhalation of chloroform was ordered when spasmodic breathing comes on.

9 o'clock, P. M. Visited the case with Dr. Thum.—Found the patient composed; face somewhat flushed; pulse about 90, full and regular; sighs frequently; mind not in the least disordered; tongue rather pale, slightly furred and moist; no thirst; no unnatural appearance about the cicatrix or arm; complains occasionally of chilliness; cannot swallow either solids or fluids, and has an evident dread of the latter, so mnch so that an enema cannot be used. No evacuation from the bowels yesterday nor to-day; complains of pain in the right shoulder, none now in the hand nor arm; but stiffness and pain in the back of the neck. The sight or sound of water produces distress, groaning and irregular spasmodic breathing. A small phial of chloroform in my hand or a sudden puff of cool air, a small piece of ice placed in his mouth, fanning, or any one, particularly a stranger, entering the room suddenly, produces groans and a gasping oppressed respiration: can perform the mere act of deglutition if there be nothing but saliva in his mouth; no desire for food; eyes have a rather unnatural, glistening appearance. Says that for five months past his skin has been subject to become very unusually dry, which made him feel miserable and very nervous until free perspiration was produced. Thinks all his present symptoms arise from playing at ten-pins for several days past. Calomel grs. x., to be placed dry on the tongue, and if it does not operate in six hours to be repeated with croton oil, gtts. ij; chloroform to be inhaled when the breathing becomes difficult, and perfect insensibility to be produced if great restlesness or general spasms come on. All exciting causes to be kept from him with special care.

July 26th, 8, P. M. Has passed a restless night, seemed to sleep once for a few minutes, but soon started up in distress. Bowels have been freely evacuated; passes urine in sufficient quantity but highly colored; pulse 80 and less full; applies frequently to his nose a handkerchief moistened with a little chloroform, which seems to speedily relieve the spasmodic breathing; face less flushed; shoulder easy but pain is felt now in the whole length of the spine. In other respects his condition is not materially changed since last evening. Blister the whole length of the spine; ung. hydr. to be rubbed into axilla and thighs, and chloroform continued as before.

1, P. M. Seems to be better. Perfect insensibility has been produced once since morning, to allay great excitement and distress produced by the door being suddenly blown open. Found him sitting on the side of the bed inhaling chloroform from the handkerchief which he holds constantly in his hand. Asks frequently for more chloroform. Pulse nearly natural; says he feels better; can take a small piece of ice enclosed in linen, in his mouth for a few moments at a time, but if continued too long his breathing becomes affected, somewhat as any one's would be by a plunge into cold water. Has had ten or twelve green evacuations from his bowels; passes urine frequently and high colored. Thinks he could swallow some mush. Treatment continued.

8, P. M. Decidedly worse. Complains greatly that persons will persist in seeing him, and is most violent in his abuse of them; entreats us to keep them away. A violent paroxysm was produced this afternoon by the presence of one whom he disliked. Complains of thirst for the first time; pulse increased in frequency; has taken about one teaspoonful of the mush; said, when I first entered the room, "oh! Doctor, I am gone!" asked for a lemon and sucked a slice of it for sometime. He is much more irritable and captious, and frequently scolds his wife and attendants. Has an especial dislike for the child, and complains that it is not kept so far away that he cannot hear it speak. Turns his face away at first, from those entering the room, and will not permit them to speak until he has spoken. When anything happens disagreeable to him, his manner becomes hurried, his respiration agitated and accompanied by groans, and he frequently exclaims, "oh, there it is, I said so." Chloroform and ung. hydr. to be continued, and sulph. morph. to be applied to the denuded surface of spine.

July 27th, 8, A. M. He is evidently much worse. Has been removed to a room on the second floor. Has been very restless, and sometimes raving through the night. Says he has eaten about half a cracker. Manifests now a fixed antipathy for several persons, particularly his wife and child, whom he will not see; says this feeling for his wife came over him suddenly last night, and implores us to give him something to stop it. Will not suffer his female attendant to speak to him even in reply. Became ungovernable for some time early this morning; kicked the foot-board of his bed furiously and tried to kick one of his attendants; seemed perfectly conscious of his condition, and asked to be confined in a strait jacket. When we arrived he became more composed, but his manner was hurried and agitated and his face and eyes have assumed a wild, highly excited and distressed expression. Talks constantly and rapidly, for the most part making complaints of his attendants and of persons and events that have been disagreeable to him during his illness. He now emits occasionally thick, frothy saliva. Much thirst, but cannot swallow anything, except so much of the ice as melts in his mouth when he applies it for a moment to his tongue. Occasionally talks cheerfully and even smiles. Breathing now almost constantly short, irregular and panting; pulse 125 and becoming weaker; skin moist. At one time says the room is hot enough to kill him, and at another complains of the chilly air. Tongue and mouth have a whitish, clammy appearance. Refuses to have the strait jacket on, and says he spoke of it this morning because he was worse and thought we had given him up. Requires all who come to his room to be announced at the door and not to speak until he first says something. When we enter the room we must take a seat at some distance from him until he choses to order us nearer, and when we approach him he always extends his hands to us: is mostly rational but occasionally his mind loses its balance: Insists that the bite of the dog did not produce his illness, but that some one has poisoned him. Continue chloroform at intervals, to produce insensibility, and apply opium cerate to the spine.

1, P. M. Symptoms still more aggravated. He is exceedingly restless and cross, and disposed to quarrel with all his attendants. Sprang from his bed and drove his female nurse in terror from the room. Spits almost continually a white, frothy saliva; hands very tremulous; free perspiration; pulse 132; respiration and deglutition as before. His dread or abhorrence of females is a very prominent symptom; will not suffer one to come near him, and commands them to be driven from the house; ordered some bonnets and other female apparel that hung on the wall, to be removed from his sight. Sends messages occasionally to his wife with but little meaning in them, but will not permit her to see him. Becoming much less manageable. He accidentally got possession of a small piece of board and without looking round threw it violently over his shoulder at some one standing behind him. Becomes highly impatient and irritated if his attendants do not answer him instantly and execute his orders with dispatch. His complaints are becoming very clamorous; is constantly ordering some one to leave the room or some one to come to him. Would not permit the cerate to be applied, and will not suffer the strait jacket to be put on; says we need not fear he will bite any of us. His looks and manner are a mixture of fear or suspicion and rage. He has not taken any fluids for now more than two days, has raging, burning thirst, but still turns from water and seems to dread it as if it were the most deadly poison. Through neglect of the attendants he happened to see several persons whom we had engaged to assist in applying the jacket, when he immediately became extremely boisterous and commenced raving at his two nurses.—Whilst we were in the adjoining room in consultation, we heard loud, piercing screams and oaths, the door flew open and the attendants rushed out in rapid retreat, followed by our patient, whose expression was surely the most horrid the human face can assume; his eyelids dilated, eye wild with rage, lips parted wide and teeth firmly clenched, arms elevated and extended before him, and every feature and line of his face expressing the most intense fury. He was immediately seized, insensibility produced by chloroform, and a strait jacket put on. A

considerable quantity of frothy saliva flowed from his mouth. In a few minutes consciousness returned and he was composed and quite rational. To be quieted with chloroform at intervals until our return.

7, P. M. More composed; talks less, but insists on having the jacket off; is less affected by cool air and the sight or sound of water; much less saliva ejected; pulse 130 and weak. Threatens us with vengeance if we do not release him; knows every one, but occasionally makes an incoherent remark. We wished him to take some ice-cream but the proposal produced distress; said he could not swallow it and would not try unless his arms were liberated. He finally consented to make the attempt; a spoonful was offered, and, with evident signs of distress, he looked steadily at it for some time and then made a sudden snatch at it with his mouth, but barely touched the spoon, turned his head convulsively away, with groans, gasping respiration and awkward efforts at deglutition. Sometimes he would thrust his tongue as far out as he could on one side of his mouth, in the direction of the spoon, and at the same time seemed to be making efforts to avert his face and eyes. Complained for the first time of pain in the abdomen, and said he would burst if the jacket were not removed. It was determined to produce insensibility with chloroform, and as consciousness began to return to give him the cream. This succeeded, and he took eight or ten teaspoonfuls which he swallowed or rather gulped down with some effort; seemed to like it, and wanted more, but as the sedative action of the chloroform wore off, would not take it. The chloroform was repeated; more cream was taken, and he asked for fried ham. At this time he was mostly rational, but made frequent threats. Injections of beef-tea; insensibility to be produced at proper intervals, and ice-cream or any other food he might be able to take, to be given.

10, P. M. Evidently dying. One injection had been given; he had taken some ice-cream and about two ounces of the ham, but soon after the latter, his attendants say, he commenced vomiting, had a slight spasm, seemed to go to sleep, but soon awoke, to die. His eyes are fixed; pupils immoveable, but neither much contracted nor dilated; breathing convulsive; tongue protruded; pulse rapidly sinks, and, without further struggle, death terminates his sufferings.

*Autopsy*, 14 *hours after death.*—Body rigid. Pharynx deeply congested throughout, the purple color ending abruptly at the commencement of the œsophagus; mucous follicles much enlarged; œsophagus unchanged; mucous coat of the stomach slightly injected, and small spots of ecchymosis in its greater extremity; stomach contained about three quarters of a pint of reddish brown fluid; the mucous membrane of the upper two-thirds of the duodenum deeply congested; that of the ileum of a pinkish hue throughout, with occasional spots of decided inflammation; the general redness extends slightly to Peyer's and the solitary glands, and the latter, near the termination of the ileum, are much enlarged and surrounded by a red areola; mucous coat of the cæcum and beginning of the colon much injected; remainder of the large bowel perfectly healthy, and contains a small quantity of dark brown, semi-fluid feces; spleen much enlarged from previous disease, but otherwise healthy. Liver perfectly natural. Kidneys gorged with blood of a venous hue. Bladder empty, and its mucous lining raised in two or three points by bloody effusion. Inferior cava distended with semi-coagulated blood. Semi-lunar ganglia perfectly healthy. Mucous membrane of the larynx, trachea and larger bronchial tubes of a deep purple hue and covered with frothy, bloody mucus. Upper lobe of the right lung much congested. Heart perfectly healthy; its left side entirely empty and the right contained a small quantity of frothy blood. Veins of the dura mater greatly distended with fluid blood. No fluid in the arachnoid sac. Substance of the cerebrum perfectly normal. Lateral ventricles contained about half an ounce of bloody serum. Veins of the surface of the cerebellum, medulla oblongata and pons varolii distended with blood. Substance of the cerebellum much less consistent than usual, but no redness. Substance of medulla oblongata and pons perfectly natural.

Time was not allowed to make a thorough examination of the spinal cord, but a section of its upper dorsal portion disclosed considerable vascular fulness of its investing membranes, and its substance to all appearance natural.

*Remarks.*—The leading characteristic of this case was high nervous excitability, and the nerves of sensation were seemingly much more affected than those of voluntary motion. This condition gradually increased in intensity until within a few hours before death. The mental faculties were mostly unimpaired, but at times, especially towards the close, they were disturbed and somewhat perverted. In my observation there were no general spasms at any time and all the voluntary muscles were perfectly obedient to the will, excepting those concerned in respiration and deglutition. The latter organs apparently, sustained the principal force of the disease; and in whatever way an exciting cause acted, whether as a disagreeable impression on the skin, or an offensive sight or sound, or an effort to swallow solids or fluids, but especially the latter, the respiratory function was the first to complain, and, in fact, seemed almost exclusively to suffer.

It is well ascertained that not more than one in twenty-five of those bitten by rabid animals ever has symptoms of hydrophobia, and this, in connection with the fact that in the present instance there had been a peculiar and unusually unhealthy, nervous state for some months preceding the accident, might suggest an inquiry, whether the virus, unaided by some morbid condition of the health, would develop the disease?

How far the morbid post mortem appearances were essential elements of the disease or were mere effects of it, modified or even produced by the treatment, I cannot pretend to determine. The highly congested condition of the pharynx, and the abrupt termination of the purple color at the œsophagus; the fact that the earliest symptoms in this case implicated this part, and that many writers mention somewhat similar appearances both in man and the dog, authorize the belief that this state of the pharynx is an essential part of the malady.

The July number of the London Lancet, for 1849, contains an account of a case of hydrophobia, in which the writer says: "The pharynx, throughout its whole extent, was of a deep, dusky red color; and the mucous follicles enlarged, so as to present, at first sight, the appearance of large aphthæ; that these were merely enlarged follicles, the scalpel proved. One circumstance requires especial notice—the abrupt and marked limitation of the congestion or inflammation, whichever it was, to the pharynx itself, between which and the top of the œsophagus the boundary was as distinct and defined as the line of separation in gangrene."

A phrenologist might perhaps be disposed to connect, in some way, the early and excessive antipathy for the child and the perfect dread or abhorrence of females, that so strangely marked the later stages, with the evidently diminished consistence of the cerebellum.

It surely cannot be that hydrophobia is necessarily a fatal disease, though the records of the profession, with fearful uniformity, seem to lead to such a conclusion.—Chemistry and Physiology will yet disrobe it of its terrors. There is a case of hydrophobia from the bite of a cat, reported in Braithwaite's Retrospect, No. xviii., in which a cure is said to have been effected by the use of chloroform. In our case a fair trial of this agent was not made on account of the absence of regular and skilful nurses; and the circumstances of the patient and the curiosity of visitors, exposed him to exciting causes, which almost incessantly counteracted the salutary sedative action of the chloroform. But so far as it was used, its effect was decidedly beneficial, allaying speedily the spasmodic breathing and producing calmness in the midst of violent paroxysms of excitement. It had the advantage of being readily and willingly inhaled, and produced all the effect we desired, when medicines by the mouth could not be swallowed, and even endermic medication was resisted. If it would do no more than enable a doomed man, as in this case, to enjoy the luxury of a few spoonfuls of ice-cream, when he is parched and consumed by thirst, and soothe the sufferings of the few hours he can live, it is a valuable remedy.

If a case of hydrophobia should now come under my care, I would insist on the most perfect seclusion, with faithful nurses and a vigilant avoidance of all exciting causes; chloroform should be used at suitable intervals to calm the nervous excitement, and appropriate local treatment directed to the condition of the pharynx.

